# Multiple Cell Cultures for MRI Analysis

**DOI:** 10.3390/ijms231710109

**Published:** 2022-09-03

**Authors:** Zuzanna Bober, David Aebisher, Marcin Olek, Aleksandra Kawczyk-Krupka, Dorota Bartusik-Aebisher

**Affiliations:** 1 Department of Photomedicine and Physical Chemistry, Medical College of Rzeszów University, University of Rzeszów, 35-310 Rzeszów, Poland; 2 Department of Orthodontics, Faculty of Medical Sciences in Zabrze, Medical University of Silesia, 40-055 Katowice, Poland; 3 Center for Laser Diagnostics and Therapy, Department of Internal Medicine, Angiology and Physical Medicine, Medical University of Silesia in Katowice, 41-902 Bytom, Poland; 4 Department of Biochemistry and General Chemistry, Medical College of Rzeszów University, University of Rzeszów, 35-310 Rzeszów, Poland

**Keywords:** cell culture, 3D cell culture, MRI

## Abstract

Magnetic resonance imaging (MRI) is an imaging method that enables diagnostics. In recent years, this technique has been widely used for research using cell cultures used in pharmaceutical science to understand the distribution of various drugs in a variety of biological samples, from cellular models to tissues. MRI’s dynamic development in recent years, in addition to diagnostics, has allowed the method to be implemented to assess response to applied therapies. Conventional MRI imaging provides anatomical and pathological information. Due to advanced technology, MRI provides physiological information. The use of cell cultures is very important in the process of testing new synthesized drugs, cancer research, and stem cell research, among others. Two-dimensional (2D) cell cultures conducted under laboratory conditions, although they provide a lot of information, do not reflect the basic characteristics of the tumor. To replicate the tumor microenvironment in science, a three-dimensional (3D) culture of tumor cells was developed. This makes it possible to reproduce in vivo conditions where, in addition, there is a complex and dynamic process of cell-to-cell communication and cell–matrix interaction. In this work, we reviewed current research in 2D and 3D cultures and their use in MRI studies. Articles for each section were collected from PubMed, ScienceDirect, Web of Science, and Google Scholar.

## 1. Introduction

Molecular imaging is used to improve diagnosis, prognosis, and monitoring of therapy in patients. Among molecular imaging methods, we distinguish optical imaging, magnetic resonance imaging (MRI), computed tomography (CT), positron emission tomography (PET), and single-photon emission computed tomography (SPECT). These methods allow for the visualization of anatomical, genetic, biochemical, and physiological changes in vivo. The development of MRI in recent years, in addition to diagnostics, has made possible implementing this method to assess the response to applied therapies. Conventional MRI provides anatomical information and, due to advances in technology, provides physiological information. Recent MRI research has focused on single cell imaging in vivo [1]. Molecular imaging technology in brain research can aid in diagnosis and treatment but can also be used to study brain function. At the molecular level, optical imaging has also been used with the support of reporter gene technology [2]. Machine learning (ML) has been implemented for cancer classification, diagnosis, genomic biomarker identification, progression detection, and survival prediction. The use of ML-based radiomic analysis allows quantification of tumor heterogeneity. It may be used in the future as a non-invasive marker for diagnosis and the monitoring of response to applied treatment as well as predicting patient prognosis. An attempt has been made to combine MRI and ML as a new tool for assessing treatment response and the prognosis of patients with high-grade gliomas (HGG) [3]. MRI is also being used to visualize stem cells (SCs) in vivo [4]. An extremely valuable advantage of this method is its non-invasive nature and the possibility for multiple studies. The implementation of contrast agents and tracers into the study allows for the detection of cells in MRI. Recent studies may also find reports from the evaluation of cellular immunotherapy using preclinical cell tracking technologies as well as the possibility of clinical translation [5]. The development of nanotherapeutics eliminates the limitations of traditional drugs and may contribute to more effective cancer therapies. Imaging modalities such as MRI, CT, photoacoustic imaging (PAI), PET, and electron microscopy [6] play key roles in monitoring drug delivery as well as controlling the therapeutic effect in real time. MR imaging can be used to decipher the delivery of albumin-targeted drugs. Many drugs inextricably bind to albumin; imaging techniques enable quantification at the molecular level and allow the assessment of mechanisms, guiding drug development and personalizing treatment [7]. A recent study evaluated the effect of a preoperative drug (POA) in pregnant women undergoing elective cesarean section. Drug levels and neonatal outcomes were evaluated, with levels of TNF-α, IL-6, IL-8, and pro-inflammatory cytokines in cord blood. Based on the analysis of the results of 66 volunteers, there was a significant correlation among POA, fetal birth weight, and the parameters of fetal cord blood TNF-α, IL-6, and IL-8. The analysis showed an increase in the levels of cytokines TNF-α, IL-6, and IL-8 in cord blood in women with high levels of POA, with negative consequences for the newborn, i.e., increased levels of inflammatory markers in fetal cord blood and significantly lower birth weight [8]. Another study presented research on the detection of severe acute respiratory syndrome coronavirus 2 (SARS-CoV-2). Researchers have developed a rapid response and quantitative capacitive aptasensor for detecting nucleocapsid protein ultrastructures based on a microelectrode array (MEA) system. Researchers have developed an MEA-based aptasensor using a specific aptamer to recognize N-protein from SARS-CoV-2 utilizing the capacity of the solid–liquid interface with picofarad-level sensitivity for detection at ultra-low femtogram per milliliter levels in various matrices. Recognition occurs at N-protein concentrations of about 10-1 ng/mL or much lower. The potential of using this method is very high for screening asymptomatic patients, which is a very good alternative to the existing method, i.e., PRC-based RNA, which is time consuming, complicated to operate, and expensive [9]. The applications of ultrasound have been widely studied in biomedical engineering. The design of a new ultrasonic transducer for future biomedical applications has great potential. The use of ultrasound transducers can be used, among other things, to disrupt the blood–brain barrier to improve drug delivery. Improving the performance of the ultrasound transducer could enable a wider use in ultrasound diagnostics, ultrasound therapy, particle/cell manipulation, drug delivery, and nerve stimulation. The invented Sm-PMN-PT monocrystal features giant piezoelectricity and a transparent PMN-PT monocrystal with ultra-high piezoelectricity. The optoacoustic transducer has the ability to precisely control the operation of the optoacoustic transducer [10]. A new, non-invasive neuromodulation integrating focused transcranial ultrasound stimulation (tFUS) offers a wide range of applications for both understanding and treating the brain. In their work, Zhang et al. described the detectability of tFUS in the application of analgesia. Based on their rat study, they deduced that tFUS stimulation with PAG can effectively suppress formalin-generated nociceptive activity. This confirms that tFUS PAG stimulation can achieve the effect of analgesia, which, in further studies, can help develop non-invasive analgesic technology [11]. Several virtual screening models for small molecules targeting primary miRNAs have been presented in research studies. Mature miRNAs and their specific target mRNAs during miRNA–mRNA interactions can form unique Argonaute-mediated functional loops (AGOs). These loops can serve as potential targets for small-molecule drug discovery. In their work, Zhuo et al. first used a loop-based and incorporated (AGO) virtual screening model to target loops. The potential of the created loop by targeting the AGO-mediated miR-214–mRNA interaction can be used to rescue the bone phenotype in genetically engineered mice [12]. The first targeted glutathione (GSH)-responsive thermostatic system (RIF@Cy5.5-HA-NG) for tuberculosis with a rifampicin (IF)-loaded near-infrared emission carrier was also presented. The hydrophobic–hydrophobic interaction induced by the photocleavage reaction was exploited, enabling the early diagnosis of TB by tracking granulomas. This allowed the selective imaging and precise inhibition of localized TB by released RIF. A gSH-activated hyaluronic acid (HA) system loaded with rifampicin was shown to achieve positive effects in TB therapy [13].

**Figure 1:**
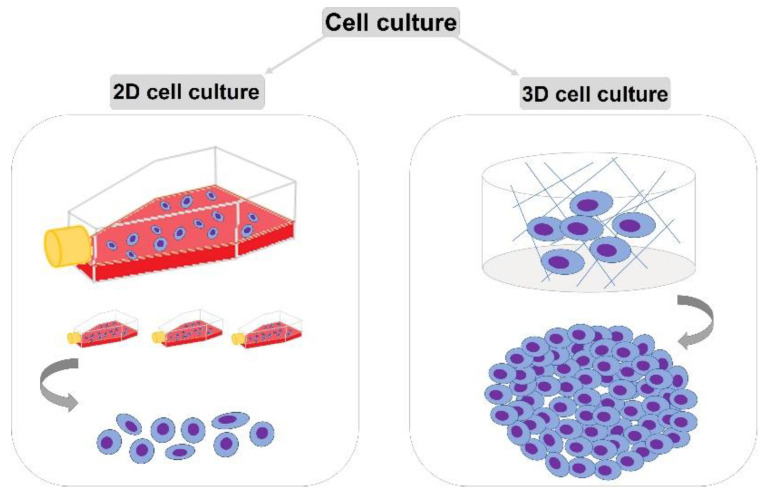
Cell culture methods: general division.

Cell culture research has a key role in scientific research including the development of imaging methods. Cell cultures enable the development of such fields of science as biotechnology, biopharmacy, or toxicology. Cell cultures are increasingly being used in pharmaceutical research for drug absorption studies and for assessing toxicity and predicting properties. The isolation and cultivation of various types of cells enables the development of various types of scientific research due to the real resemblance to a living organism. Currently, two methods of cell culture are used: two-dimensional (2D) and three-dimensional (3D) (Figure 1). In classic 2D cell culture, cells multiply in monolayers on a previously prepared medium. The 2D culture method has many limitations and does not allow for full control of cell growth and differentiation. The medium used plays an extremely important role in cell culture because it provides the cells with the necessary nutrients, growth factors, and hormones and the antibiotic content protects the cells and regulates the pH. Growth rate is usually logarithmic. To achieve this growth phase, pass the cells regularly. After the culture reaches about 80% confluence, transfer them to sterile bottles with fresh medium. In the case of divided adherent cells, after the removal of the culture medium, most often the cell layer is “trypsinized”. After washing the monolayer with, 0.9% NaCl, 0.25% EDTA trypsin solution is added in an appropriate amount. The cells are then incubated under the same conditions as used for culture after about 3.5 min under microscopic control. The cell suspension is then added to the culture medium in an appropriate volume, which, after mixing, can be transferred to new and sterile culture vessels.

Cell cultures conducted in laboratories require special conditions: appropriate humidity, temperature, and gas concentration in the incubator must be maintained. In laboratory conditions, it is possible to control the conditions and create an optimal microenvironment for breeding. In addition, we have the option of applying active substances to breeding, such as, for example, growth factor, hormones, or proteins. In addition, cell culture should always be carried out under sterile conditions, maintaining full sterility of the room and all tools and substances used for the cultivation. The culture vessels must also be sterile and disposable. All materials used for the cell culture should be non-toxic to cells and allow for their growth and gas exchange with the environment. A 2D cell culture media can be made, for example, of polystyrene (PS) [14] or glass [15]. Growth media are used for breeding; the complete medium is most often enriched with heat-inactivated fetal bovine serum (FBS) and an antibiotic. The cultivation is normally carried out in an incubator with the following parameters: temperature 37 °C, 95% air, 5% CO_2_, and 100% relative humidity. The appropriate growth medium is selected depending on the cultured cell line. The 2D cell cultures ensure a low cell concentration and, moreover, do not correspond to the tumor morphology in vivo. The implementation of a 3D culture enables the reconstruction of the tumor microenvironment and the development of a procedure for conducting a 3D culture with high repeatability and preserving the tumor features and allows for the implementation of preclinical tests. The historical development of 3D breeding methods is presented in Table 1. Many physicochemical and biochemical factors influence the cells in the natural environment, which, in turn, influences the proliferation, reproduction, and development. The implementation of 3D breeding enables the re-creation of natural conditions. The 3D cultures involve many methods; for example, the use of 3D cell cultures in a bioreactor allows for an extremely high density of cells that grow in a controlled environment. These types of cultures enable high repeatability and the possibility of carrying out research, e.g., on medicinal substances. Compared to the monolayer culture, the 3D cell culture better reflects the tumor structure. In the 3D model, we have an interaction between adjacent cells and the extracellular matrix (ECM). While these interactions affect the biochemical and mechanical signals of cell physiology, there is no such interaction for cells grown in 2D. In contrast, in the case of a 3D culture, we can create an environment by mimicking in vivo conditions. With the use of 3D methods, we obtain more information about the interactions between cells: the model that creates a tumor reflects its metabolism and characteristics.

**Figure 2:**
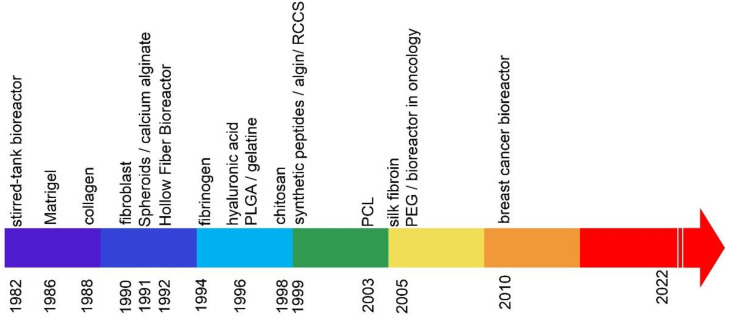
Years of publications on hydrogel-based 3D matrices for cultivation.

On the other hand, in 3D cultures, various methods are used, including those based on hydrogels, polymers, and glass fibers, forming the so-called “scaffolds”. The years of publications on 3D matrices based on a hydrogel for cultivation are shown in Figure 2. 

However, in the case of scaffold-free techniques, we can find microplates with hanging droplets, magnetic levitation, and spheroidal microplates with a coating with a very low level of adhesion. Nodular models are widely used as avascular tumor models for metastasis and for screening. Breeding techniques using different types of scaffolding have many advantages. They can be used to build spheroids [34], which we prepare depending on the needs and the experiment being conducted. Spheroids are a type of three-dimensional cell cultures of many cell lines that have the ability to self-organize [35]. An exemplary model is the multicellular spheroid (MCS) model, which reflects the similarity to real tissues in many respects.

### 1.1. Nodular Cell Culture

One example of a 3D cell culture is the nodular culture. This type of culture shows limited adhesion to the substrate, which allows the clusters of cells to form into three-dimensional clusters of many cells, which allows the three-dimensional contact of the cells. Moreover, it should be noted that these types of cultures have a limited diffusion of nutrients into the inner part of the spheroid; therefore, the spheroids in their volume are varied. Inside, there are cells with necrotic changes, followed by cells in the resting phase, and, in the last few layers, cells that are proliferating. Due to their structure, spheroids are used as models of non-vascularized neoplastic tumors used, for example, to assess the effectiveness of anticancer drugs. For example, Foxall presented a 3D nodular culture model of diffuse large B-cell lymphoma (DLBCL), with the replication of tumor microenvironment (TME) components consisting of malignant and benign cells, including CAF and tumor-associated macrophages (TAMs) that drive tumor initiation and tumor growth. Based on the research and analysis, it was found that the viability of DLBCL cells grown in the 3D system, compared to 2D, is characterized by a higher viability [36]. Haro and Orsulic, in their work, demonstrated that the DLBCL stromal gene signature shows a greater chance of survival in DLBCL and several other B-cell lymphomas, in contrast to ovarian cancer [37]. Diffuse DLBCL rebuilds the fibroblastic mesh network, reprogramming the HLFs into CAFs, which acquire the ability to modulate TME. CAF activation increases PD-L1 expression, which results in abnormal immunosuppressive abilities [38]. In addition, the humoral factor pyruvate, which is secreted, inter alia, by the patient’s cancer-associated fibroblasts, promoted cell survival for primary lymphoma cells [39]. Kuen et al. presented a 3D model of a pancreatic cancer (PC) cell culture with CAF and monocytes to evaluate cellular mechanisms in the tumor immunosuppressive microenvironment. Immunosuppressive cytokines were produced that induce M2 polarization and also demonstrated the ability to inhibit CD4+ and CD8+ T cell activation and proliferation in in vitro studies [40]. In their research, the group of Dolznig et al. grew human colon tumor cells as multicellular spheroids and then cultured them together with normal fibroblasts or CAF in collagen I gels [41]. In addition, CAF mediated inflammation in human breast and ovarian tumors through the pro-inflammatory factors IL-6, COX-2, and CXCL1 [42].

### 1.2. The 3D Culture Matrices Based on a Hydrogel

The 3D cell cultures using hydrogels are increasingly being carried out. These are highly hydrated hydrophilic polymers. Hydrogels form a network of fibers with a specific pore size to reflect the extracellular matrix. Due to the fact that they are transparent, hydrogenation makes it possible to observe the cell culture. Moreover, their great advantage is the fact that they have a good diffusion of nutrients. Hydrogels used in 3D breeding can be natural or artificial [43]. The natural ones are most often based on natural polymers, for example, fibrinogen [44], hyaluronic acid [45], collagen [46], Matrigel [47], gelatin [48], chitosan [49], and alginate [50]. In addition, hydrogels based on the extracellular matrix (ECM), due to soluble factors (growth and cytokines) implanted into the stroke cavity, can attract endogenous cells; the migration of cells to the ECM hydrogel enables tissue regeneration [51]. Hydrogels are more and more often superior to 2D cultures due to the fact that they turn out to be more physiological. In stem cell research, 3D hydrogel-based cultures of cells grow in a manner similar to the in vivo situation. Depending on the composition, the hydrogels may be more or less hydrated, and, thus, may have a different flexibility, which also affects the mechanical properties of the scaffold. Another advantage is that by changing polymer concentrations or cross-linking methods, we can change the physical properties of the 3D model. In addition, in vitro studies on 3D models improve mapping to the tumor in vivo, due to the fact that cells do not have a homogeneous growth environment and are exposed to a lack of sufficient oxygen and nutrients inside the tumor, which may affect tumor progression [52]. Moreover, it may be of importance in the case of the supply of pharmaceutical substances to cell cultures. In a 2D culture, pharmaceuticals such as anticancer agents reach cells without barriers. However, the situation in the in vivo environment is different. Therefore, research on 3D cell cultures turns out to be important. Tumor cells grown on 3D matrices show significant features of native tumor tissues. It turns out that the tumor structure significantly changes the diffusion profile of the drug. In the literature, we can read many reports on the description of the physicochemical properties of native micro-neoplastic environments. Various types of interactions play an important role: cell–cell interactions and cell matrix [53]. Various types of 3D models have the features of solid tumor tissues with a corresponding morphology. Xu et al., in their work, presented the mutual interactions between the tumor and its stroma. A two-layer hydrogel system based on hyaluronic acid (HA) for tumor culture from LNCaP (PCa) prostate cancer cells was developed and characterized. There was a significant increase in the expression of two pro-angiogenic factors, vascular endothelial growth factor-165 (VEGF (165)) and interleukin-8 (IL-8), both at the mRNA and protein levels [54]. In addition, in the literature, we can find many reports on the creation of matrices for the 3D breeding of natural and synthetic origins. Among the natural matrices, we can distinguish matrices derived from fibroblasts, Matrigel™ or Cultrex^®^ [55], type I collagen, silk fibroin, alginate, or hyaluronic acid. On the other hand, among the synthetic matrices, we most often find hydrogels based on poly (ethylene glycol) (PEG) [56], poly (lactide-co-glycolide) (PLGA) [57], scaffolds made of electrospun poly (ε-caprolactone) (PCL) [58], or synthetic peptides [59].

## 2. Natural Matrices

### 2.1. Culture Based on Matrigel™ or Cultrex^®^

On the other hand, Matrigel™ and Cultrex^®^ are extracts from basement membranes, consisting of type IV collagen, laminin, perlecan, etc., used, inter alia, for human colon adenocarcinoma SW480 [60], human breast adenocarcinoma MDA-MB-231 [61], and pancreatic adenocarcinoma Capan-1 [62]. Wessels et al., on the basis of a 3D breast cancer cell culture, demonstrated the complex signaling between fibroblasts and breast cancer cells, which coordinates aggregation and coalescence, which is a specific response of 3D cells [63]. The study presented a 3D model of an MCF-7 breast cancer cell culture based on Matrigel and an assessment of the survival of MCF-7 transfectants expressing wild-type or catalytically inactive ST3 (ST3wt or ST3cat-) [64]. Kasper et al. found that the overexpression of stromelysin-3 exacerbated the tumor in MCF-7 and MDA-MB-231 cell cultures in an orthotopic model of human tumor xenograft in nude mice and in a Matrigel-based 3D culture system [65]. On the other hand, Song et al., in their research, used 3D collagen gel and Matrigel to evaluate the expression and reorganization of structural proteins of rabbit aortic smooth muscle cells [66]. Matrigel hydrogel in combination with a synthetic polymer of polylactic and co-glycolic acid (PLGA) and polylactic acid (PLLA) was also used for 3D lung tissue engineering [67]. Li et al. described the positive effect of the 3D Matrigel environment on pig tooth enamel cells [68]. In other studies, researchers demonstrated 3D culturing of stem cells under pulsed conditions [69].

### 2.2. Collagen-Based Matrices

Collagen biomaterials are widely used as 3D scaffolds for cell culture [70]. Due to the fact that it is the main component of the extracellular matrix (ECM), type I collagen is used for the cultivation of human breast cancer MCF-7 [71], human hepatocellular carcinoma HepG2 [72], and human epithelium of the small intestine [73]. A recent study by Quarta et al. presented the composition of agarose–collagen hydrogels and spheroid cells of three different breast cancer cell lines, MCF-7, MDA-MB-361, and MDA-MB-231 [74]. Rossi et al. assessed the effect of HSF fibroblasts on the growth of MCF-7 cells and their sensitivity to radiation in collagen gels. Growth inhibition and increased sensitivity to radiation were found [75]. Moreover, the models of the cultures on 3D collagen scaffolds caused a differentiated morphology of cells, which is related to the porosity of the scaffold, and also a prolonged cell proliferation. Additionally, the use of 3D collagen scaffolds can generate a population of cells with cancer stem cell (CSC) properties [76].

### 2.3. Matrices Derived from Fibroblasts

In the case of matrices derived from fibroblasts, they are used as a microenvironment in advanced cancers of, for example, the colon [77] or pancreas [78]. Models based on fibroblasts show a different cell morphology compared to 2D cultures. However, despite many advantages, it should be noted that this type of breeding is associated with a longer preparation and differences in the composition of individual batches. Moreover, matrices based on fibroblasts may not fully reproduce the structure and composition of the tumor microenvironment [79]. In addition, fibroblasts have an influence on the tumor initiation capacity. In contrast, cancer-associated fibroblasts (CAFs) play a role in the adhesion and motility of tumor cells. Moreover, they are considered to be the most abundant type of stromal cells in various cancers and constitute a heterogeneous population of cells. They stimulate tumor growth and progression in many types of cancer. In addition, CAFs secrete H_2_O_2_, stimulating cancer through the stroma and transformation of primary epithelial cells and intensification of the aggressiveness of cancer cells. Liao et al., in their work, presented the effect of cancer-associated fibroblasts on tumor growth and metastasis in a 4T1 model of murine breast cancer. Studies have shown that modulating the immune microenvironment influences both tumor growth and metastasis [80]. Additionally, Yavuz et al. emphasized in their work that CAFs play a key role in carving the tumor microenvironment in breast cancer. Fibroblasts help induce immunosuppressive PD-1 + TAM [81]. CAFs are also responsible for the induction of FGF4 expression in ovarian cancer stem cells [82]. Fibroblasts are considered to be the most abundant cells in the connective tissue proper. They cause the production of collagen, various growth factors, cytokines, and chemokines and the degradation of the extracellular matrix. It should be emphasized that cancer-related fibroblasts cause the migration and invasion of cancer cells [83] and contribute to driving tumor growth [84]. Human fibroblasts are also involved in carcinogenesis, proliferation, and metastasis of A-549 non-small cell lung cancer. Studies have shown that CAF, compared to normal fibroblasts (NF), increases the invasion of A549 cells, as demonstrated by Horie et al. based on a 3D model of in vitro culture [85]. Additionally, Nakamura et al. conducted a 3D model to evaluate the effect of podoplanin-positive fibroblasts on tumor cell proliferation. Studies have shown that proliferation increases with lung cancer [86]. In contrast, immunofibroblasts support the earliest stages of the formation of tertiary lymphoid structures (TLS) [87]. Recent studies by Cribaro et al. analyzed the complexity of the microenvironment of glioblastoma (GBM). The group presented a 3D visualization showing the exact composition of human GBM in the form of a three-dimensional landscape of the GBM vascular microenvironment [88]. On the other hand, the group of Chhetri et al., in their work, presented an important step in cancer research based on the evaluation of a 3D cell culture. The influence of the microenvironment of a 3D culture on the cell nucleus and its influence on the stimulation mechanisms of the nucleus were presented [89]. An important aspect in cell culture is also the influence of electrical biostimulation and silver ions on fibroblast cells. The group of Zhao et al., in their latest publication, assessed porcine fibroblast cells and changes in the dynamics of the cell transcriptome after electrical biostimulation in the presence of silver ions. After analysis, no increase in the expression of pluripotency genes was found [90].

### 2.4. Matrices Based on Calcium Alginate

An equally frequently used biodegradable hydrogel is alginate [91], which has valuable properties because it has a controlled pore size and does not adhere to cells. Alginate-based hydrogels are used as 3D scaffolds [92,93]. In addition, a covalently cross-linked hydrogel provides chemical stability [94]. Alginate is useful in the culture of human hepatocellular carcinoma MHCC97L, HCCLM3 [95], and oral squamous cell carcinoma OSCC-3 [96]. Cavo et al. developed a new alginate–Matrigel hydrogel for use in the 3D culture of aggressive human breast cancer cells (MDA-MB-231), which perfectly mirrors the tumor in vivo [97]. Chen et al. presented porous 3D scaffolds made of Ca alginate in the form of a human osteoblast (hOB) platform and calcium alginate scaffolds. In addition, bioreactors were constructed for the transplantation of human osteoblast clusters [98]. Using the MCF-7 breast cancer cell line and a 3D cell culture model based on alginate hydrogels, the assessment of cell activity depending on the flexibility of the medium was made. Studies have shown that the number of MCF-7 cells decreased as the flexibility of the hydrogel-based medium increased. It was noted that in the most delicate hydrogels, cell proliferation was highest after 2 weeks [99]. Karimpoor et al. used alginate foam for a 3D culture; it allowed them to obtain a porous scaffold that perfectly reflects the structure of the bone marrow (BM). Moreover, it increased bone marrow differentiation in both leukemia and hematopoietic cells [100]. In other studies, Shakibaei et al., using a 3D model of an alginate-based scaffold, investigated the effects of curcumin on HCT116R cells and chemotherapy based on the drug 5-fluorouracil (5-FU), which confirmed the use of the model, which was also used in the study of drug effects [101]. Additionally, Utech et al., using hydrogels, developed mesenchymal stem cells (MSCs) in the microspheres, which allowed them to obtain a continuous supply of nutrients that caused the growth and proliferation of aspirated cells [102]. Sidhu et al. presented research in which they used a 3D platform based on alginate microcapsules. It was shown to allow the study of hESC proliferation and differentiation into different lines [103]. Xu et al., using a 3D dynamic culture, investigated the effect on the regeneration of cartilage tissue in rabbit joints using encapsulated cells [104].

### 2.5. Matrices Based on Fibrinogen

Almany and Seliktar, in their work, described a hydrogel scaffold based on fibrinogen and polyethylene glycol. The advantages of this type of scaffolding were presented, including high plasticity while maintaining biological functionality [32]. Additionally, Dikovsky et al. described the use of a synthetic polyethylene glycol (PEG) scaffold and endogenous fibrinogen precursor molecules, demonstrating the preservation of quality between the molecular architecture of the matrix and the cell morphology [105]. On the other hand, Kim et al., due to the fact that the mechanical properties of PEG–fibrinogen-based hydrogels decrease significantly over time in PBS, found a positive effect of ascorbic acid on the improvement of matrix properties [106]. On the other hand, Shachaf et al. developed a biomimetic material based on fibrinogen and PluronicF127 [107]. In contrast, Pradhan et al., in their work, presented PEG–fibrinogen hydrogels for the 3D culture of breast cancer of three cell lines: MCF-7, SK-BR-3, and MDA-MB-231 [108].

### 2.6. Matrices Based on Hyaluronic Acid

In addition, there are reports in the literature on the use of hyaluronic acid (HA) for the cultivation of three-dimensional tumor models, which is structurally an ideal equivalent of in vivo conditions. Hyaluronic acid is an essential component of ECM in the tumor microenvironment used in human prostate cancer [109] and human glioblastoma U87MG [110,111]. It has the following properties: it is involved in the formation of blood vessels within the tumor and its metastasis and also interacts with cell surface receptors such as CD44 or RHAMM. Recent studies by Harris et al. presented biocompatible 3D scaffolds decellularized from plant material using carbon dioxide. The cultivation of human fibroblast cells on the surface of a spinach leaf demonstrated the biocompatibility of decellularized scCO_2_ scaffolds [112]. On the other hand, in their research, Feng et al. cultured MCF-7 breast cancer cells in 3D using fibrous polycaprolactone (PCL) scaffolds, showing an increased proportion of CSC [113].

### 2.7. Gelatin-Based Matrices

Jiang et al. presented, in their work, an alginate/gelatin composite hydrogel with adjustable mechanical and adhesive properties. As a result, it is possible to obtain mechanically soft gels with a larger number of cell adhesion groups or stiffer with a smaller number [114]. Additionally, Jiang et al., in another study, presented the use of an alginate/gelatin hydrogel with breast cancer cells and fibroblasts, forming a 3D model [115]. On the other hand, Wang et al. used bacterial cellulose–gelatin hydrogels for the breast cancer cell line (MDA-MD-231) as a 3D scaffold. Studies showed that cells exhibited significant adhesion, proliferation, ingrowth, and differentiation [116].

### 2.8. Chitosan-Based Matrices

Dhiman et al. presented a chitosan-based matrix for the cultivation of MCF-7 breast cancer cells. The results showed that glucose consumption and melate production were similar to cell growth in tissue culture flasks, which indicated that it can be used for the 3D cell culture of the MCF-7 line [117]. Additionally, Dhiman et al., in their previous studies, used these matrices to determine the cytotoxicity of tamoxifen. Studies showed the effect of tamoxifen on estrogen-positive cancer cells and the inhibition of cathepsin D uptake from the culture medium [118]. In the latest published studies, the group of Mohseni et al. described chitosan–alginate polymer nanoparticles that were synthesized as a clinical Dotarem^®^ carrier based on (Gd 3+) used for labeling mesenchymal stem cells for MRI imaging in in vitro studies [119]. In their work, Huang et al. assessed the influence of spatial architecture on the behavior of cells in 2D and 3D cultures based on chitosan scaffolds [120].

### 2.9. Alginate-Based Matrices

Another example of a natural matrix is an alginate-based matrix. Lan and Starly presented a platform for the 3D culture of liver cells with high density and single-layer growth of the breast cancer cell line (MCF-7), which allowed for the assessment of the drug dose concentration and the effect on breast cancer cells [121]. On the other hand, the group of Yu et al. used alginate core-shell beads to grow MCF-7 breast cancer 3D cells [122]. Conversely, Lee et al. investigated human breast cancer cells expressing GFP (GFP-MCF-7) encapsulated in an alginate hydrogel in a 3D model. The culture was assessed using a capacitive sensor, which made it possible to monitor the migration of human mesenchymal stem cells (hMSC) [123].

### 2.10. Matrix Based on Silk Fibroin

Another natural material is silk fibroin, which has good biocompatibility and unique mechanical properties, which allows it to be used in 3D models [124]. It is a matrix used, inter alia, for the cultivation of human breast adenocarcinoma MDA-MB-231 [125] and breast cancer EMT6 [126]. Chaturvedi et al. used 3D silk fibroin scaffolds to differentiate human skeletal muscle cells’ myoblasts in vitro [127]. Ashari et al. investigated silk fibroin for the properties of improving the condition of mouse islets in vitro against inflammatory stress during islet cell transplantation [128]. On the other hand, Lovett et al. used silk fibroin for microvessels on a microvascular scale (ID < 6 mm). Research has shown that the use of silk fibroin-based microducts can be used as a biomaterial for microvascular transplants [129]. In other studies, Zhao et al. used apatite-coated silk fibroin scaffolds in conjunction with bMSC to repair jaw injuries in dogs [130]. In contrast, other studies by Wang et al. presented hybrid nanofiber scaffolds made of silk fibroin (SF) and poly (lactide-co-ε-caprolactone) (PLCL) with improved properties for bone regeneration [131]. Niu et al. presented silk fibroin labeled with quantum dots and their interactions with cells used for controlled drug delivery [132]. Other studies presented an in vitro 3D tumor model of breast cancer from the MDA-MB-231 cell line on a silk fibroin scaffold to evaluate the efficacy of cyclosaplin; studies showed a significant decrease in MMP-9 activity in the tumor [133]. The group of Bhardwaj et al. used scaffolds made of a mixture of silk fibroin and chitosan for the engineering of cartilage in in vitro studies [134]. An earlier study by Mauney et al. used silk fiber biomaterials together with collagen and polylactic acid scaffolds for 3D scaffolds in adipose tissue engineering. They presented 3D silk fibroin scaffolds with human bone marrow and adipose-derived mesenchymal stem cells [135]. In addition, silk fibroin was used to create 3D scaffolds to repair damage to articular cartilage. It turns out that during cultivation, the initial density of cell seeding is important: the denser the cell seeding is, the faster the rate of formation of functional cartilage tissue can be [136]. Additionally, the team of Li et al. used silk fibroin as well as chitosan and gelatin to culture 3D MC3T3-E1 cells in bone tissue engineering. Four types of scaffolds were prepared: SF, SF/C, SF/gel, and SF/Cs/gel. After analysis, they concluded that the best choice would be the SF/Cs/gel scaffold [137].

## 3. Synthetic Matrices

### 3.1. Poly (Lactic-Co-Glycolic Acid) PLGA Matrices

PLGA porous, biodegradable synthetic scaffolds are also used on a large scale, mainly for oral squamous cell carcinoma OSCC-3 [138] and human glioblastoma U251 [139] but also for bone marrow mesenchymal stem cells [140]. By contrast, Sahoo et al., in their research, presented the characteristics of the porous, biodegradable PLGA/PLA microparticles used for the 3D culture of breast cancer [141]. Priwitaningrum et al. presented a 3D nodular matrix reflecting the tumor stroma and performed penetration tests of silica and PLGA nanoparticles [142]. Published papers also include reports on 3D PLGA scaffolds used for tissue engineering [143].

### 3.2. Synthetic Peptides

Synthetic peptides with a defined amino acid composition for easy incorporation of specific biological ligands are also used for 3D culture, for example, in SK-OV-3 human ovarian cancer [144] or MCF-7 breast cancer [145]. Synthetic polymers, on the other hand, are used for 3D printing of organs. Biodegradable synthetic polymers have very good mechanical properties and are made of non-toxic products [146]. Fong et al. created a 3D model of Ewing’s sarcoma ex vivo based on porous three-dimensional scaffolds made of electrospun poly (ε-caprolactone). Studies have shown that this type of scaffold can be used to evaluate new anticancer drugs for mechanistic bone sarcomas [147].

### 3.3. Dies Based on Electrospun Poly (ε-Caprolactone) PCL Scaffolding

Biologically neutral synthetic PCL scaffolds [148] are also popular, used, inter alia, for breeding Ewing’s sarcoma TC-71 [134], prostate cancer PC3 [149], and LNCaP [150]. In a recent study by Zhao et al., they presented the culture of human gingival fibroblasts (HGF) on poly (lactide and glycolide) (PLGA) scaffolds and assessed the effect of Smad4 on caspase-3 and Bcl-2 expression [151]. On the other hand, Hamedani et al., in their work, presented an innovative PLGA-based electrospun polymer fiber scaffold for renal cell carcinoma (RCC). The scaffold was loaded with a natural compound showing a strong anticancer and anti-inflammatory effect, which is Honokiol, which showed an inhibitory effect on the proliferation and migration of kidney cancer cells [152]. In turn, Zheng et al., in the latest research, presented the 3D scaffold HA15 bone tissue, printed in 3D β-TCP/PLGA technology with the drug promoting osteogenesis HA15 in in vivo studies in rabbits. This type of scaffold has been shown to be a good substitute material for the treatment of bone defects [153].

### 3.4. Matrices Based on Poly (Ethylene Glycol) (PEG)

In the case of synthetic matrices, PEGs with controlled biochemical and mechanical properties are most often used. They are used, inter alia, for the cultivation of human ovarian epithelial carcinoma OV-MZ-6 [154] and human pancreatic ductal adenocarcinoma PANC-1 [155]. Balion et al., instead, presented studies on human HROG36 glioma, C6 glioma cells in rats, and human A37 melanoma cells [156]. Lee et al., on the basis of their research, found that semi-penetrating network compositions based on PEG–diacrylate and hyaluronic acid can support the survival, spread, and migration of 3D cells [157]. Additionally, in their latest research, McKee and his group analyzed naive human embryonic stem cells (ESCs) grown on PEG-based 3D scaffolds. Based on the research, the possible mechanisms of self-renewal of naive ESCs were presented for the first time [158]. On the other hand, viscoelastic hydrogels enable the regulation of stress relaxation, which, as it turns out, after studies by Nam et al., has an important role in cell differentiation, spread, and proliferation. Alginate–PEG hydrogels coupled with RGD are characterized by a faster relaxation, which enables the spread and proliferation of fibroblasts and increased osteogenic differentiation of MSCs [159]. Su et al., in their work, presented an overview of applications of scaffolding printed in various 3D technologies made of various raw materials. The presented scaffolds can be used in regenerative medicine using stem cells for bone tissue engineering [160]. On the other hand, the group of Hassan et al. presented 3D cultures of human adipose tissue-derived stem cells encapsulated in a hydrogel composed of a hyperbranched copolymer based on PEG and hyaluronic acid. Based on the research, the secretion of growth factors for wound healing in the PEG–hyaluronic acid (HA) hybrid hydrogel was assessed [161]. Urrios et al. presented the developed PEG-DA-250 print on a transparent, low-molecular-weight poly (ethylene glycol) diacrylate resin for cell culture, which can be used for culture for several days [162]. Kutikov et al., in their work, presented biodegradable scaffolds based on PEG for tissue engineering [163]. On the other hand, Nemeth et al. used nano-sighted PEG-GelMA-HA hydrogels for three-dimensional chondrogenic dental pulp stem cell (DPSC) spheroids, which enabled chondrogenic differentiation in in vitro studies [164]. Cruz-Acuña et al. used PEG–4MAL hydrogels to encapsulate human pluripotent stem cells’ (hPSC) HO into damaged mouse colon in in vivo studies. Research confirmed that PEG–4MAL promotes HO implantation and accelerates colon wound healing [165]. Fernandes-Cunha et al. used a type I collagen hydrogel of cross-linked polyethylene glycol (PEG)-N-hydroxysuccinimide (NHS) and assessed its regenerative capacity in the case of corneal defects in in vitro and in vivo tests [166].

## 4. The 3D Cell Culture

The use of a 3D cell culture in research allows the mimicking of physiological vascular networks and the engineering of organs and tissues in vitro [31]. As pre-presented in Table 1, this is one of the bioreactors that allow a 3D culture. Special attention should be paid to the features of this type of bioreactor, mainly, the excellent mass transport properties. HTB bioreactors allow the transport of nutrients without restriction by diffusion, which is often a limitation in the creation of large in vitro constructs.

### 4.1. Hollow Fiber Bioreactors

Hollow fiber bioreactor-type bioreactors are also used for cell cultures, which are used in numerous preclinical applications, including the cultivation of monoclonal antibodies and growth hormones, and also in 3D cell cultures. It is a bioreactor that perfectly reflects the in vivo environment. In these types of bioreactors, nutrients are delivered to the cells in a controlled manner, while the metabolic components are utilized. These types of bioreactors are suitable for culturing for an extended period of time, depending on the selected cell line. The main components that make up this type of bioreactor are the intragranular (IC) and extra-spherical (EC) spaces. Cells are seeded into the EC space of the fiber bioreactor and grow there. The cell culture medium is pumped through the IC space to provide oxygen and nutrients. The use of a bioreactor with capillary fibers allows us to obtain a very large number of cells in a small volume. The cell culture can fill the EC space to a density >10^8^ cells/mL. Compared to the 2D culture in the bioreactor, we have a 10-fold increase in the number of cells. In addition, this type of bioreactor enables the continuous exchange of nutrients and the exchange of metabolic products. In addition, the fiber in this type of bioreactor is porous and it turns out to be a very good substrate for cell attachment. In addition, the culture parameters are strictly controlled and constant; we do not have any changes in temperature, pH, or other biochemical changes and the influence of mechanical stress, which may also be important in a cell culture. A recent study by Gobin et al. presented a culture in a hollow fiber bioreactor with extracellular vesicles (EV) produced by bone marrow-derived mesenchymal stromal cells (hBM-MSC). The production of EV from hBM-MSC with immunoregulatory components was carried out in the same quantity and with the same quality for a longer period [167]. It should also be noted that EVs contain many bioactive molecules that can affect intercellular communication, including stromal cell re-education, changing the microenvironment, and tumor metabolism and metastasis, and may even increase drug resistance. Tai et al., in an article, presented the role of extracellular vesicles in prostate cancer [168].

### 4.2. Stirred-Tank Bioreactors (STBR)

Another type of bioreactor is the stirred-tank, which is used in chemical and environmental engineering. A work by Rodriguez-Granrose et al. demonstrated the transition from static culture to a stirred-tank bioreactor. The study assessed the production of beads in the Erlenmeyer system and of discogenic cells for the development of cell therapy for the treatment of lumbar disc degeneration [169]. On the other hand, Manstein et al. combined STBR with the power of in silico process modeling for the best results in a human pluripotent stem cell (hPSC) culture [170].

### 4.3. Rotary Cell Culture System (RCCS) Bioreactors

Rotary cell culture system bioreactors are also used for three-dimensional microgravity cultures. Zheng et al., in their work, assessed the proliferation of MCF-7 cells cultured in rotary cell culture systems (RCCS). The studies showed that this type of culture, by inducing the ERK1/2 pathway, can promote the proliferation of MCF-7 cells [171]. In contrast, Fournier and Harrison used RCCS to study MLO-Y4 osteocytes in collagen–hydroxyapatite scaffolds [172]. Cui et al., in their work, said that RCCS increases NTRK3 expression in an NSC cell culture grown in the RCCS system on a collagen sponge [173].

## 5. Conclusions

Carrying out cell cultures requires special precautions, as they are very often pathogenic, which is why biological safety is so important. In addition, it is important to maintain aseptic conditions so that the culture is not contaminated with microorganisms. Cell cultures are grown in an optimally selected medium. The emergence of three-dimensional cell cultures in in vitro studies is closer to in vivo structures. It is possible to conduct, e.g., drug interaction studies and applied therapies that were not possible in the case of single-layer cultures. The 3D cell culture enables many experimental studies such as drug research, cell physiology evaluation, therapy evaluation, and tissue engineering.

## Figures and Tables

**Table 1:** Historical development of 3D breeding methods.

Cell Culture Method	No.	Type of Cell Culture		References
**3D cell culture**	1	Spheroidal		[16]
	**3D culture matrices based on a hydrogel**			
	2	**Natural Polymers**	Matrigel	[17]
	3		Collagen	[18]
	4		Fibroblast	[19]
	5		Calcium alginate	[20]
	6		Fibrinogen	[21]
	7		Hyaluronic acid	[22]
	8		Gelatine	[23]
	9		Chitosan	[24]
	10		Algin	[25]
	11		Silk fibroin	[26]
	12	**Synthetic Polymers**	Poly (lactic-co-glycolic acid) (PLGA)	[27]
	13		Synthetic peptides	[28]
	14		Scaffolding made of electro-spun poly (ε-caprolactone) (PCL)	[29]
	15		Poly (ethylene glycol) (PEG),	[30]
	16	**3D bioreactors**	Hollow fiber bioreactor	[31]
	17		Stirred-tank	[32]
	18		Rotary cell culture system (RCCS)	[33]
